# The Metabolome-Wide Signature of Major Depressive Disorder

**DOI:** 10.21203/rs.3.rs-3127544/v1

**Published:** 2023-09-21

**Authors:** Rick Jansen, Yuri Milaneschi, Daniela Schranner, Gabi Kastenmuller, Matthias Arnold, Xianlin Han, Boadie W Dunlop, Mood Disorder Precision Medicine Consortium, A John Rush, Rima Kaddurah-Daouk, Brenda WJH Penninx

**Affiliations:** a Amsterdam UMC location Vrije Universiteit Amsterdam, Department of Psychiatry, Amsterdam, the Netherlands; b Amsterdam Public Health, Mental Health Program, Amsterdam, the Netherlands; c Amsterdam Neuroscience, Mood, Anxiety, Psychosis, Sleep & Stress Program, Amsterdam, the Netherlands; d Institute of Computational Biology, Helmholtz Zentrum München, Neuherberg, Germany; e Department of Psychiatry and Behavioral Sciences, Duke University, Durham, NC, USA; f Barshop Institute for Longevity and Aging Studies, University of Texas Health Science Center at San Antonio, San Antonio, TX, USA; g Department of Psychiatry and Behavioral Sciences, Emory University School of Medicine, Atlanta, GA, United States.; h Department of Psychiatry and Behavioral Sciences, Duke University School of Medicine, Durham, Durham, NC, USA.; i Duke National University of Singapore, Singapore; j Department of Medicine, Duke University, Durham, NC, USA; k Duke Institute of Brain Sciences, Duke University, Durham, NC, USA

## Abstract

Major Depressive Disorder (MDD) is an often-chronic condition with substantial molecular alterations and pathway dysregulations involved. Single metabolite, pathway and targeted metabolomics platforms have indeed revealed several metabolic alterations in depression including energy metabolism, neurotransmission and lipid metabolism. More comprehensive coverage of the metabolome is needed to further specify metabolic dysregulation in depression and reveal previously untargeted mechanisms. Here we measured 820 metabolites using the metabolome-wide Metabolon platform in 2770 subjects from a large Dutch clinical cohort with extensive depression clinical phenotyping (1101 current MDD, 868 remitted MDD, 801 healthy controls) at baseline and 1805 subjects at 6-year follow up (327 current MDD, 1045 remitted MDD, 433 healthy controls). MDD diagnosis was based on DSM-IV psychiatric interviews. Depression severity was measured with the Inventory of Depressive Symptomatology self-report. Associations between metabolites and MDD status and depression severity were assessed at baseline and at the 6-year follow-up. Metabolites consistently associated with MDD status or depression severity on both occasions were examined in Mendelian randomization (MR) analysis using metabolite (N=14,000) and MDD (N=800,000) GWAS results. At baseline, 139 and 126 metabolites were associated with current MDD status and depression severity, respectively, with 79 overlapping metabolites. Six years later, 34 out of the 79 metabolite associations were subsequently replicated. Downregulated metabolites were enriched with long-chain monounsaturated (P=6.7e-07) and saturated (P=3.2e-05) fatty acids and upregulated metabolites with lysophospholipids (P=3.4e-4). Adding BMI to the models changed results only marginally. MR analyses showed that genetically-predicted higher levels of the lysophospholipid 1-linoleoyl-GPE (18:2) were associated with greater risk of depression. The identified metabolome-wide profile of depression (severity) indicated altered lipid metabolism with downregulation of long-chain fatty acids and upregulation of lysophospholipids, for which causal involvement was suggested using genetic tools. This metabolomics signature offers a window on depression pathophysiology and a potential access point for the development of novel therapeutic approaches.

## Introduction

Major Depressive Disorder (MDD) is a multifactorial disorder with high disease burden and chronicity in many patients. The pathophysiology of MDD is complex, with substantial molecular alterations and dysregulations of multiple pathways. Metabolomic technologies capturing simultaneously hundreds of molecules may provide a comprehensive reading of depression pathophysiology. A review [1] summarizing metabolomic analyses in depressed patients using urine, cerebrospinal fluid and blood samples showed that metabolites implicated in energy metabolism and neuronal integrity and transmission were altered. In a large-scale pooled analysis using the lipidomics Nightingale platform in 10,145 controls and 5283 depressed cases [2], we identified a metabolic profile (21 metabolites) for lifetime depression characterized by a shift towards less HDL and more triglycerides and glycoprotein acetyls, these findings were replicated in the recent UK biobank analysis using the same platform [3]. Such findings not only indicate pathway dysregulations that contribute to depression symptomatology development, but also help explain why comorbidities like metabolic syndrome [4], obesity [5], diabetes [6]) and cardiovascular disease [7] occur more often in depressed than non-depressed persons.

While targeted metabolomics platforms are limited by design and often overrepresented by a certain class of metabolites (like lipids on the Nightingale platform), untargeted platforms cover a larger portion of the metabolome and have the potential to uncover previously unrecognized pathobiological mechanisms. In a population-based study, the untargeted Metabolon platform was used to measure 353 unique metabolites in serum of 1411 subjects [8]. Participants with elevated depressive symptoms assessed with a self-report scale (N=72), had decreased levels of two acylcarnitines involved in mitochondrial fatty acid oxidation. A larger pooled analysis of population-based cohorts (Amin *et al., submitted,* N~13,000), showed that self-reported symptoms of depression were associated with 8 metabolites directly derived from food or products of host/microbial food-derived products. Nevertheless, previous studies did not select patients diagnosed using psychiatric interviews and likely included relatively few participants with major depression. Furthermore, metabolites were assessed only at a unique time-point, thus precluding the possibility of replicating the metabolite-depression associations.

The association between metabolite concentrations and depression identified in observational studies may emerge from different causal pathways: 1) shared factors (e.g., lifestyle, medication use) impacting both metabolite levels and presence of depression; 2) reverse causation of subclinical depression impacting on metabolite levels via behavioral changes (e.g., reduction in physical activity, worsening of dietary habit); and 3) direct causal action of metabolite alterations on depression pathophysiological pathways. Mendelian Randomization (MR), a causal inference technique leveraging genetic variants as proxy instruments whereby random allele segregation limits confounding and reverse causality, can help to disentangle cause and consequence in the metabolite-depression associations. For instance, in previous MR studies, we showed that alterations in inflammatory pathways [9] and acylcarnitine metabolism [10] may have a potential causal role for the development of depression, while this was not supported for omega-3 fatty acids [11].

In the current study, we measured 820 metabolites using the Metabolon platform in subjects from a large Dutch clinical cohort (N=2770) with extensive clinical phenotyping conducted at baseline and at 6-year follow up. The unprecedented sample size, broad metabolite spectrum, and longitudinal data provides a reliable, state-of the-art metabolite signature of depression. Furthermore, we applied MR analyses to examine the nature of metabolite-depression associations leveraging results from the most recent and largest GWAS on Metabolon platform [12](N~14,000 and depression [13] (N~800,000).

## Methods

### Study design and participants

Data were from the Netherlands Study of Depression and Anxiety (NESDA), an ongoing longitudinal cohort study examining course and consequences of depressive and anxiety disorders. The NESDA baseline sample consists of 2,981 persons between 18- and 65-years, including persons with a current or remitted diagnosis of a depressive and/or anxiety disorder (74%) and healthy controls (26%). Individuals were recruited from mental health care settings, general practitioners, and the general population in the period from September 2004 to February 2007. Persons with insufficient command of the Dutch language or a primary clinical diagnosis of other severe mental disorders, such as severe substance use disorder, or a psychotic disorder, were excluded. Participants were assessed during a 4-hour clinical visit, consisting of the collection of all somatic and mental health determinants in the current study, as well as a fasting blood draw. Similar face-to-face assessments were repeated after two, four, six and nine years [14]. The current study uses metabolomics data from 2770 participants (2463 from NESDA baseline and 307 siblings newly recruited at the 9-year follow up, pooled as discovery cohort) and 1805 participants of the 6-year follow-up (of which 1685 overlapped with baseline and were used for replication analyses). Persons with an anxiety disorder without MDD were excluded from the analysis. The NESDA study was approved by the Ethical Review Boards of participating centres, and all participants signed informed consent. More than 94% of the NESDA participants were from North European origin. The population and methods of the NESDA study have been described in more detail elsewhere [14, 15].

### Metabolite measures

After an overnight fast, EDTA plasma samples were collected and stored in aliquots at −80°C until further analysis. Samples were send in two batches to the USA. Metabolic profiles were measured using the untargeted metabolomics platform from Metabolon Inc (Durham, NC). Briefly, plasma samples were divided into four fractions; two for ultra-high performance liquid chromatography-tandem mass spectrometry (UPLC-MS/MS; positive ionization), one for UPLC-MS/MS (negative ionization), and one for a UPLC-MS/MS polar platform (negative ionization). Peaks were quantified using the area-under-the-curve in the spectra. To account for run-day variations, peak abundances were normalized by their respective run-day medians. Compounds were identified using an internal spectral database.

### Metabolite QC

The raw metabolite data set included measures of 5181 samples and 1008 reference samples (well-characterized human plasma samples) which were used to calculate and control for technical measurement variability with in total 1367 metabolites measured in 29 batches. One experimental sample and one reference sample with a high missingness (> 5SD + mean missingness) were excluded from the dataset. We further excluded metabolites with a missingness in over 30% of all samples. If outliers or apparent measurement issues within one plate or several plates within one batch were observed, all values on that plate were set to ‘NA’ to not affect subsequent batch correction. We batch corrected data by normalizing all samples to the batch median and then excluded those metabolites that still had a technical measurement variability of >30%. Next, we imputed missing metabolite measures. Before imputation, we tested if missingness in any of the remaining 820 metabolites accumulated in one of the three measurement waves (baseline, 6 or 9 year follow up) using a Fisher’s exact test. As this was not the case, we jointly imputed all waves using a k-nearest neighbor approach (k=10) [16]. Before statistical analysis, we log2 transformed the final dataset. For each metabolite, outliers larger than the mean plus five standard deviations were set to the mean plus five standard deviations. Outliers smaller than the mean minus five standard deviations were set to the mean minus five standard deviations.

### Clinical assessment and covariates

Presence of current MDD (i.e., occurring within six months prior to the interview) was assessed by the DSM-IV Composite International Diagnostic Interview (CIDI) version 2.1. Depression severity levels in the week prior to assessment were measured with the 28-item Inventory of Depressive Symptomatology (IDS) self-report [17]. Several covariates were included in the models assessing the associations between metabolites and depression. Alcohol consumption was assessed as units per week and smoking status was coded as current, ex- and never-smokers. Physical activity was assessed using the International Physical Activity Questionnaire (IPAQ) [18] and expressed as overall energy expenditure in Metabolic Equivalent Total (MET) minutes per week (MET level * minutes of activity * events per week). Body mass index (BMI) was calculated as measured weight divided by height squared. The number of self-reported current somatic diseases for which participants received medical treatment was counted. We used somatic disease categories as categorized previously [19, 20]: cardiometabolic, respiratory, musculoskeletal, digestive, neurological and endocrine diseases, and cancer. Educational level was measured as years of education. Antidepressant use was based on drug container inspection of all medications used in the past month, classified according to the World Health Organization Anatomical Therapeutic Chemical classification, and included selective serotonin reuptake inhibitors (ATC code N06AB), serotonin–norepinephrine reuptake inhibitors (N06AX16, N06AX21), tricyclic antidepressants (N06AA) and tetracyclic antidepressants (N06AX03, N06AX05 and N06AX11).

### Instrument selection for Mendelian randomization

Summary statistics for metabolomics measured with the same untargeted mass spectrometry-based platform (Metabolon HD4) were retrieved from a GWAS including up to ~14,000 samples [12] (interrogation of the mGWAS results can be performed at www.omicscience.org). The Psychiatric Genomics Consortium performed an overarching meta-analysis [13] of all available GWAS datasets with depression phenotypes including established MDD diagnosis or self-declared depression, totalling 246,363 cases and 561,190 controls. Lack of sample overlap across the two discovery GWAS reduced the likelihood of MR estimates biased toward the observational correlation [21]. Metabolites GWAS summary statistics were processed removing non-SNP variants, strand-ambiguous SNPs and those with MAF<1%. Variants overlapping and allele-matching with those reported in depression GWAS were clumped (10,000 kb window, *r*^*2*^=0.001, EUR population of 1000Genomes used as linkage disequilibrium reference) to identify independent significantly (p<5.0e-8) associated SNPs.

### Statistical analyses

For each of the 820 metabolites measured at the NESDA baseline wave, we ran a GEE model with the metabolite as dependent, and depression status or depression severity (total IDS) as the independent variable, while correcting for education, sex, age, physical activity, smoking status, alcohol use, number of chronic diseases and shipment (samples were shipped in 2 batches), and using family ID as clusters. Depression status was coded as 3 level factor (controls, remitted MDD, current MDD), using the controls as reference. BMI was added to the model in additional analysis. Effects of antidepressants (AD) were verified by recomputing the associations between MDD status and metabolites while removing the antidepressant users. For metabolites associated with depression status and severity, that did not show much stronger associations with AD use as compared to the associations with MDD status, analysis computing associations with depression outcomes were repeated using the 6-year follow up data. We consider the baseline effects replicated if nominal P-values in the follow-up data for depression status or severity were smaller than 0.05, and directions of effect were consistent. In the 6-year follow-up sample no family relations were present so instead of GEE, linear models were used, with the same covariates as assessed at the 6-year follow-up. Enrichment analysis was done using pathways pre-assigned to the metabolites by Metabolon (Table S1). Two classes of pathways were assigned, 10 super pathways and 95 sub pathways. As only 681 metabolites were classified in one of the pathways, this set was used as the reference in the enrichment analysis. Enrichment between significant metabolites and each pathway was computed by applying the Fisher exact test to the contingency table.

### Mendelian randomization

For the metabolites identified in baseline data and replicated at the follow up, and for which at least two independent associated SNPs were found in the corresponding GWAS, two-sample Mendelian randomization (2SMR) analyses based on GWAS summary statistics were performed to test the potential causal role of metabolites on lifetime depression risk. For each metabolite, genome-wide significant independent SNPs used as instruments were aligned on the positive strand for exposures (metabolites) and outcome (depression). 2SMR analyses were performed based on the inverse variance weighted estimator [22]. False discovery rate (FDR) *q*-values according to Benjamini-Hochberg procedure were calculated taking into account multiple testing. The robustness of significant results was tested in sensitivity analyses based on weighted median [23] and MR-Egger [24] estimators. Furthermore, heterogeneity among included SNPs was tested via Cochran’s Q test, single SNP, and leave-one-out SNP analyses. The presence of potential horizontal pleiotropy (a genetic instrument for exposure influencing the outcome by mechanisms other than exposure) was tested using the MR-Egger intercept [25] and the MR-PRESSO method [26]. Analyses were conducted using the R MR-Base package [27].

## Results

### Demographics

Metabolites from the Metabolon platform (N=820) were measured in whole blood samples from 2770 participants (2463 recruited at NESDA baseline and 307 siblings recruited at 9-year follow up). Participants had current MDD (N=1101), remitted MDD (N=868) or were healthy controls (N=801), 65% female, mean age was 43 years (sd 13) and mean BMI was 26 (sd 5, Table 1).

### Cross sectional associations between metabolites and depression status and severity at baseline

For each metabolite, a GEE model was fit (correcting for family structure) using MDD as dependent variable (MDD as 3 level factor, control group as reference) while correcting for demographics and technical covariates (see Methods). When comparing controls and persons with current MDD, 139 metabolites were significantly different (Table S1, FDR<5%, 92 metabolites downregulated, 47 upregulated). Most significant was methylstearate (beta=−0.26, P=5.6e-9, FDR=4.5e-6), downregulated in the current MDD group. There were 88 metabolites significantly different between the remitted and control groups (86 downregulated), 57 overlapped with the metabolites associated with current MDD. Betas of the 139 metabolites associated with current MDD, were strongly correlated with the betas from the association with remitted MDD (Figure S1, r=0.81, 99% of downregulated metabolites had same direction of effect, 68% of upregulated metabolites had same direction of effect), but most effects were stronger for current MDD (65%). When adding BMI to the models, 133 of the 139 metabolites were still significantly associated with MDD. For each metabolite, a linear model was fit in the total sample using the total IDS score as measure of depression severity as dependent variable while correcting for demographics and technical covariates. There were 126 metabolites associated with depression severity (FDR<5%, 67 upregulated), 79 of them overlap with those found for current MDD (57% of 139 hits). From the 139 metabolites associated with current MDD, the betas were strongly correlated with the betas from the association with depression severity (Figure S2, r=0.89, 99% of downregulated metabolites with same direction of effect, 100% of upregulated metabolites with same direction of effect).

### Pathway enrichment

From 10 super pathways assigned to the metabolites, the 79 metabolites associated with current MDD and depression severity included 42 lipids (significantly enriched, P=7.2e-4). From 95 sub-pathways, the 39 metabolites downregulated in current MDD were overrepresented with long-chain monounsaturated fatty acids (P=2.8e-4), long-chain saturated fatty acids (P=2.8e-4) and Hemoglobin and Porphyrin Metabolism (P=6.7e-4). The 40 metabolites upregulated in current MDD were overrepresented with lysophospholipids (P=1.3e-4, Table 2, Table S2 for full overview).

### Effects of antidepressants

From the 1101 participants with current MDD, 466 were using antidepressants (302 used SSRIs, 42 used TCAs, 122 used SNRIs). To evaluate whether identified associations between metabolites and MDD status were driven by antidepressant use, associations between metabolites and MDD status were computed on the sample without the 466 participants that used antidepressants. From the 79 identified metabolites that were associated with current MDD and depression severity, the betas from the analysis without antidepressant users correlated strongly with the betas from the analysis including antidepressant users (Figure S3A, r=0.89, 99% of betas in the same direction) showing that the identified effects were not only present solely due to antidepressant use. To compare effects caused by antidepressant use and those associated with MDD status, associations between metabolites and SSRI, TCA or SNRI use were computed (Table S1). Inspection of effect sizes (Figure S3B-C) showed only one outlier (5-methylthioadenosine (MTA)) with > 2* stronger effect of TCA compared to current MDD, this metabolite was removed in further analysis.

### Replication of findings at 6-year follow up wave

**Figure 1.**
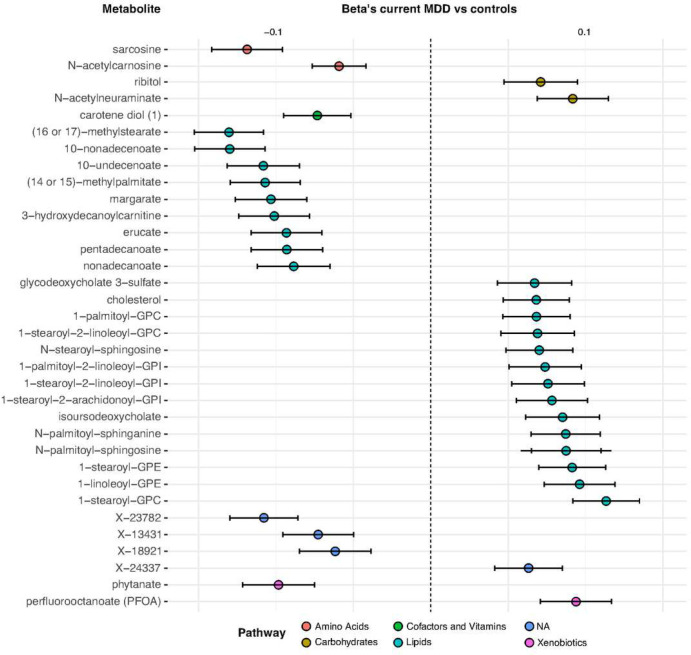
Forest plot of the 34 replicated metabolites (standardized betas for the associations with current MDD at baseline).

We aimed to replicate the 78 metabolites associated with current MDD and IDS at baseline, and that were not strongly associated with AD use, using 6-year follow-up data. In 1805 respondents (1685 overlap with baseline participants) the same metabolite measures were done in whole blood samples (controls (N=433), remitted MDD (N=1045), current MDD (N=327)). For each metabolite, a linear model was fitted using MDD as dependent variable (MDD as 3-level factor, control group as reference) while correcting for demographics and technical covariates. For 34 metabolites, the nominal P-value for the association with current MDD or depression severity was smaller than 0.05 and the directions of effects were consistent between baseline and follow up analysis (Table 3, Figure 1). The betas of the 139 metabolites that were associated with current MDD at baseline, were strongly correlated with the same betas from 6-year follow up (Figure S4A, r=0.64). A similar finding was present for the 126 metabolites associated with depression severity at baseline (Figure S4B, r=0.78) suggesting general consistency of findings.

### Mendelian randomization analyses

For the 34 replicated metabolites, 20 had GWAS summary statistics available and at least two independent associated SNPs. F-statistics (all F>10, Table S3) indicated that the strength of selected genetic instruments was adequate [28]. Table 4 shows 2SMR IVW estimates of depression risk (expressed as odds ratios [ORs] and 95% confidence intervals [95%CIs]) per SD increase in genetically-predicted log-transformed metabolite levels. An increased risk (OR=1.09, 95%CIs=1.05–1.13) of depression was significantly (q=2.1e-4) associated with genetically-predicted higher levels of 1-linoleoyl-GPE (18:2) (Figure S4). Sensitivity analyses confirmed the robustness of this result: causal estimates obtained via weighted-median (OR=1.09, 95%CIs=1.05–1.13) and MR-Egger (OR=1.09, 95%CIs=0.98–1.21) estimators were completely in line with those from IVW analyses. Additional analyses did not show statistically significant evidence of heterogeneity (Cochran’s Q *p*=0.20) or horizontal pleiotropy (MR-Egger intercept *p*=0.98; MR-PRESSO global test *p*=0.45) across SNPs indexing 1-linoleoyl-GPE (18:2) (single SNP MR Figure S6A; leave-one-out SNP MR Figure S6B). Finally, we performed a PheWAS (phenome-wide association scan) using the GWAS ATLAS Resource [29] to examine the association with other traits of the instrument top SNP (11:61569830, rs174546), which is located in the highly pleiotropic 3’-UTR region of FADS1 (fatty acid desaturase enzyme). The PheWAS (Table S4) reported significant GWAS association with a wide array of metabolic (e.g., fatty acids, cholesterol, triglycerides), cardiovascular (e.g., heart rate), immunological (e.g. red cell, platelet) or psychiatric (e.g., sleep duration, irritability) traits.

## Discussion

We presented the largest untargeted metabolomics depression study to date in a clinical sample with two measurement waves. This allowed the identification of new metabolites, not previously associated with depression, for which the effects remained consistent six years later. Around half of the identified metabolites were lipids, showing a specific system of lipid upregulation and downregulation in depression involving lysophospholipids and long-chain fatty acids. The other half consisted of metabolites previously not targeted by the mostly lipid-based metabolite platforms and are part of a wide range of pathways discussed below. Using genetic data a causal metabolite effect on depression was confirmed.

The effects of the 139 metabolites associated with the current MDD group were also apparent in the remitted MDD group, with a remarkable similarity in effect sizes in particular of the downregulated metabolites. These seemingly persistent downregulated metabolites are largely unaffected by the state of depression. They could be either consequences (scars) of illness or potentially antecedents of illness onset. The 79 metabolites associated with current MDD and depression severity were 53% lipids but also covered 7 out of 9 super pathways and 39% of all sub pathways, indicating a dysregulation of metabolites in depressed patients that does not only concern lipids but a wide spectrum of metabolites. The two pathways enriched in these 79 metabolites were both lipid pathways: lysophospholipids were upregulated and long chain fatty acids (both monounsaturated and saturated) were downregulated in the current MDD group. These pathways have been shown to be inter-connected by common chemical steps controlled by genetic variation within the fatty acid desaturase (FADS) locus on chromosome 11 (11q12.2–q13.1, containing the genes FADS1, FADS2 and FADS3 genes) [30]. This fatty-acid transforming metabolic pathway is responsible for the synthesis of over 100 individual PUFA- and LC-PUFA-containing phospholipid and lysophospholipid molecular species that have been differently linked to innate immunity, energy homeostasis, brain development, and neurocognitive functions [31–33].

Lysophospholipids have not been identified in relation to depression before in large-scale studies, because they are not targeted by most lipid-based platforms. Out of 16 measured lysophospholipids, the 6 associated with current MDD all cluster with intercorrelations between 0.32 and 0.78. Lysophospholipids activate specific G-protein-coupled receptors and are regulators of cell growth and survival, cell to–cell contacts and adhesion, and Ca2+ dependent functions. Thereby, lysophospholipids play a role in development of the nervous system, cancer growth and inflammation [34]. Reports on both rodent models and human plasma with small sample size (N<120) showed higher lysophospholipids in depression as compared to controls [35–38]. The lysophospholipids we identified are not well known, however two of the best-characterized lysophospholipids, lysophosphatidic acid and sphingosine-1-phosphate, are crucial in neurodegenerative diseases, especially in Alzheimer’s disease [39]. Dysfunction of these metabolites can lead to accumulation of amyloid-β peptides, neurofibrillary tangles and neuroinflammation [40, 41]. From these two metabolites, only sphingosine-1-phosphate was measured by the Metabolon platform we used, but it did not show an association with depression.

Two recent large-scale studies using the Nightingale platform [2, 3] consistently reported lower total cholesterol, and higher triglycerides, saturated fatty acids and monounsaturated fatty acids (MUFA’s) in patients with lifetime MDD as compared to controls. A comparison between these two studies and ours is not straightforward due to the differences in design (population-based sample vs clinical sample) and the difference in platforms (triglycerides and component measures of total cholesterol are not measured by the Metabolon platform, and the Nightingale platform does not measure any individual fatty acids and only provides accumulative measures of the MUFA’s). The downregulation of monounsaturated fatty acids (MUFAs) and saturated fatty acids we observed in current MDD is not in line with these two large studies [2, 3]. However, all measured polyunsaturated fatty acids (PUFAs) were lower in patients with depression in our study, which was similarly found in the UKBB and NESDA studies [3, 42]. There is a lot of debate about the possible role for PUFA’s in depression and in particular the prevention of depression with inconsistent findings in the literature. A large-scale meta-analysis of observational studies found a decrease of the PUFA omega 3 associated with depression [43], but a meta-analysis of prevention studies using omega 3 did not show promising results [44]. Recent work suggests many trials of PUFAs may have used doses too low to have a significant antidepressant or preventive effect, with 4 grams/day of eicosapentaenoic acid have both positive effects on both clinical and inflammatory outcomes [45, 46].

Two recent studies evaluated metabolites measured with the Metabolon platform in relation to depression. A population-based study [8], *N*=1411 of whom 72 had self-reported depressed mood) identified lower levels of the medium-chain acylcarnitine laurylcarnitine in depressed subjects as compared to controls. Consistently, in the present study the medium-chain acylcarnitine 3-hydroxydecanoylcarnitine was negatively associated with MDD. Interestingly, a previous genomic-based study using MR showed that altered metabolism of the medium chain acylcarnitines octanoylcarnitine and decanoylcarnitine is potentially causal for the development of depression [10]. While measures of laurylcarnitine, octanoylcarnitine and decanoylcarnitine were not available in the present study, it is important to note that acylcarinites of similar chain length share a substantial proportion of their genetic liability, as indicated by strong genetic correlations between them [10]. A pooled analysis of population-based cohorts (Amin *et al., submitted, N*~13,000), reported 53 metabolites associated with self-reported symptoms of depression from which 43 were also measured in our study, and 17 were replicated. From the 126 metabolites associated with depression severity in our study, Amin et al. measured 96, and 36 were replicated (37.5%), and from the top 15 hits 53% was replicated (*P*<0.05, with consistent directions of effect, Sup. Table S5). The fatty acid findings from our study were partially replicated (47% with P<0.05) and had overall consistent effect sizes (73%) but some lysophospholipid findings had opposite directions of effect.

The association between depression and the metabolomic signature identified may arise from different, non-mutually exclusive, causal pathways. Shared factors (e.g., lower socioeconomic status, presence of chronic somatic diseases, use of medications) may impact both metabolite levels and depression. However, adjustment for major sociodemographic, lifestyle and health-related factors had a marginal impact on the associations identified. A relevant confounding role is expected for BMI, whose inclusion in the statistical models partially reduced the strength of the association between depression and metabolites. However, BMI-adjusted estimates should be carefully interpreted due to the complex causal pathways between BMI, metabolite levels and depression. BMI and adiposity in general may indeed represent a confounder, a mediator, but also a consequence (collider) of metabolic alterations affecting both metabolite concentrations and depressive symptoms. Nevertheless, as opposed to previous large-scale studies using the Nightingale platform [2], after additional statistical adjustment for BMI the majority of associations detected in the present study remained statistically significant. Similarly, the use of antidepressant has a limited impact on the metabolite-depression associations, as shown by substantially similar estimates for all metabolites obtained in subjects not using antidepressants. Only the level of one metabolite, 5-methylthioadenosine, was strongly connected to the use of tricyclic antidepressants. In another causal scenario, depression impacts on metabolite levels via behavioral symptoms: for instance, worsening of dietary habit may reduce the intake of fatty acids included in metabolites pathways found to be downregulated in depression, such as long-chain monounsaturated and saturated fatty acids. In the opposite scenario, alterations in metabolite metabolism may have a direct causal action on depression pathophysiology. In a previous genomic study leveraging Mendelian randomization techniques, we showed that alteration in metabolism of medium-chain acylcarnitines, involved in fatty acids transport into mitochondria for b-oxidation, may have a potential causal role for the development of depression [10]. In the present study, the medium-chain 3-hydroxydecanoylcarnitine was found among the metabolites more consistently associated with depression across different measures and assessments.

Leveraging summary statistics from large-scale GWA-studies, we applied Mendelian randomization in the present study to examine the potential causal role in depression onset of 20 metabolites (13 lipids, 2 carbohydrates, 2 amino acids, 1 cofactor/vitamin and 2 unidentified). Results from Mendelian randomization suggest that higher levels of 1-linoleoyl-GPE (18:2), or the mechanism translating genetic variation to higher levels of this metabolite, are potentially causally involved in the development of depression. 1-linoleoyl-GPE (18:2) is a lysophospholipid, part of the cluster of lysophospholipids mentioned above. The precursor of this lysophospholipid is 18:2 fatty acid which is comprised of two isomers with double bond locations at n-6 and n-9 positions, respectively. We recognize that the n-6 isomer represents the majority of this precursor and is largely uptaken from dietary resource [47]. However, the n-9 isomer of this fatty acid involves the activity of desaturases such as FADS1. Therefore, similarly to other PUFA-containing lysophospholipids, phospholipids, free fatty acids and endocannabinoid, the metabolism 1-linoleoyl-GPE (18:2) is tightly regulated by the FADS1 (fatty acid desaturase) gene. The genetic instrument indexing 1-linoleoyl-GPE (18:2) includes the SNP rs174546 in the 3′-UTR regulatory region of FADS1. Findings from a recent genomic sequencing study showed that this variant was the top signal in the association between the FADS locus and 52 lipids containing fatty acids [30]. Consistently, the wide-range examination of previous GWAS results (pheWAS) performed in the present study showed that rs174546 is associated with a wide array of metabolic, cardiovascular, immunological or psychiatric traits. This biological complexity suggests caution in interpreting MR results using instruments including variants from the FADS locus that could index different metabolites. A previous MR-based study suggested a potential causal role in depression for polyunsaturated fatty acids such as omega-3, indexed by FADS SNPs [48]. This conclusion is in contrast with different large-scale RCTs showing no effect of omega-3 supplementation in the prevention of depression [49, 50]. Rather than attributing the complex FADS genetic signal to a specific metabolite, a more cautious interpretation of MR results points to a potentially broader involvement in depression pathophysiology of FADS-regulated metabolic processes, which may be involved in various metabolic, cardiovascular and immunological functions. Deeper mechanistic studies are needed to disentangle the specific metabolite effector relevant for depression.

The strength of our study is that it is the largest study to date, using data from two time points, with untargeted metabolomics and deep psychiatric characterization with different measures of depression including clinically established diagnoses of MDD, as opposed to previous studies using a population based sample. Limitations are that although partially replicated in a similar previous study (Amin *et al., submitted)* and internally replicated at another time point in a largely overlapping sample, many of the identified metabolites will need to be confirmed in other large samples, ideally with similar clinical assessments. The cross-sectional design of our study did not permit drawing inferences on causality, besides the MR finding. Furthermore, estimates from MR describe a potential average lifetime causal effect unable to distinguish specific critical windows or acute events. Also, genetic instruments were derived from GWAS based on samples of participants of European ancestry, so results cannot be generalized to different populations.

In conclusion, this study provides novel and confirms previously established metabolite pathways involved in depression. The untargeted whole-metabolome approach shows that a wide range of metabolites is dysregulated in depression, involving various metabolites interconnected around the FADS metabolic pathways. This may represent the biological substrate connecting depression with different cardiometabolic health outcomes. Furthermore, genomics-based analyses suggested a potential causal involvement of this pathway in the pathophysiology of depression. This metabolomic signature is a promising target for treatment. Nevertheless, future work is required to unravel the exact causal mechanisms across the complex network or processes around the FADS pathways through further mechanistic studies.
